# Flexible Fusion Structure-Based Performance Optimization Learning for Multisensor Target Tracking

**DOI:** 10.3390/s17051045

**Published:** 2017-05-06

**Authors:** Quanbo Ge, Zhongliang Wei, Tianfa Cheng, Shaodong Chen, Xiangfeng Wang

**Affiliations:** 1 School of Automation, Hangzhou Dianzi University, Hangzhou 310018, China; qbge@hdu.edu.cn (Q.G.); ctf811412@163.com (T.C.); 2 State Key Laboratory of Management and Control for Complex Systems, Institute of Automation, Chinese Academy of Sciences, Beijing 100190, China; 3 School of Computer Science and Engineering, Anhui University of Science and Technology, Huainan 232001, China; 4 Science and Technology on Electro-Optic Control Laboratory, Luoyang Institute of Electro-Optical Equipment of Avic, Luoyang 471000, China; Chen-shao-dong@163.com; 5 Shanghai Key Lab for Trustworthy Computing, East China Normal University, Shanghai 200062, China; xfwang@sei.ecnu.edu.cn

**Keywords:** flexible fusion structure, mixed fusion method, combinatorial optimization, sensor subsets selection, tracking accuracy, system survivability

## Abstract

Compared with the fixed fusion structure, the flexible fusion structure with mixed fusion methods has better adjustment performance for the complex air task network systems, and it can effectively help the system to achieve the goal under the given constraints. Because of the time-varying situation of the task network system induced by moving nodes and non-cooperative target, and limitations such as communication bandwidth and measurement distance, it is necessary to dynamically adjust the system fusion structure including sensors and fusion methods in a given adjustment period. Aiming at this, this paper studies the design of a flexible fusion algorithm by using an optimization learning technology. The purpose is to dynamically determine the sensors’ numbers and the associated sensors to take part in the centralized and distributed fusion processes, respectively, herein termed *sensor subsets selection*. Firstly, two system performance indexes are introduced. Especially, the survivability index is presented and defined. Secondly, based on the two indexes and considering other conditions such as communication bandwidth and measurement distance, optimization models for both single target tracking and multi-target tracking are established. Correspondingly, solution steps are given for the two optimization models in detail. Simulation examples are demonstrated to validate the proposed algorithms.

## 1. Introduction

The rapid development of some key technologies—for example, communication technology, sensor technology, data processing, and so on—have promoted the research into applications of wireless sensor network systems. According to different demands, there are mainly two types including passive and active networks. Due to their different working principles, the data processing methods are clearly different. Therefore, we should have different views when designing data fusion algorithms for sensor networks. Actually, it is necessary that fusion algorithms should have adaptive function because of the complex application background. Namely, the fusion structure of the task network system should be flexible. This means that the task network system can dynamically determine and choose the sensors taking part in the fusion processes. Namely, the flexible fusion also indicates that sensor numbers are time-varying for centralized and distributed fusion methods. Then, most of the traditional fusion methods cannot be directly used to deal with dynamical sensor selection. To some extent, the sensor selection belongs to the sensor management domain. It is important and significative to study flexible fusion methods for wireless task network systems.

Multisensor fusion is a fundamental technique for networked information systems (NIS), which can fuse different kinds of measurement data from multiple sensors. It has the advantage of reducing the uncertainty of target perception and improving the performance of the NIS [1,2,3,4,5,6]. For instance, designing multi-sensor data fusion algorithms in order to improve target tracking learning system performance has recently been one of the popular topics in the NIS area. The traditional fusion tecnologies mainly include centralized and distributed fusion methods. The two approaches have different performance and application background. Accordingly, it is a good way to combine the two fusion approaches—the mixed fusion structure with the centralized and distributed fusions is more effective for task network systems. Thereby, the issue of how to ensure that the sensors to respectively take part in the centralized fusion and distributed fusion is important and challenging. In addition, the adopted basic nonlinear filter also affects the fusion performance for nonlinear systems. An improved self-adaptive unscented Kalman algorithm was presented in [7] to ameliorate the stability of target tracking. A particle filter algorithm is proposed based on optimizing the dynamic neighborhood self-adaptive particle in order to raise target tracking accuracy in [8]. Compared with the traditional Kalman filter, a fixed gain Kalman filter which was studied in [9] had better performance from the perspective of computational cost. Further, based on the self-adaptive neuro-fuzzy inference system, In [10], Ma et al. presented an improved Kalman filtering algorithm to reduce the tracking error. Sun et al. [11] presented an improved extended Kalman filter-based target tracking learning algorithm. However, overall research on the sensor fusion structure is still lacking; until recently, most works were mainly based on single and fixed fusion methods. They have not considered the case that an initial sensor fusion structure would not be adaptive to the whole tracking process because of the richness of the target tracking system, while simultaneously lacking a sensor fusion structure recombination design. The focus of this paper is mainly to design and solve an optimization model on sensor subsets selection, so only the traditional unscented Kalman filter (UKF) is used. In fact, the nonlinear filters mentioned above can be used in our optimization solution to improve associated fusion estimation and tracking performance.

In this paper, the flexible fusion structure concept is introduced to improve the universality of the learning system. Based on our early work in [12], we will explain the concept, formation, and application scenes of the flexible fusion structure in detail, and further analyze the advantages of flexible fusion structure relative to the fixed fusion structure. Here, a fixed fusion structure means that once the fusion method (e.g., centralized fusion or distributed fusion) is determined, neither the fusion method nor the sensors taking part in the fusion process are changed during the whole working time. Clearly, this approach does not satisfy the practical requirements of engineering applications. However, because the flexible fusion structure has a dynamical adjustment function, it has better self-adaptive adjustment ability than the fixed structure. Additionally, it can quickly and flexibly regulate the system resources allocation to respond to the change of the tracking situation. Aiming at the above conditions and based on the earlier work [12], we focus on this popular research topic (i.e., flexible fusion structure for target tracking or state estimation). In order to achieve self-adaptive adjustment ability, some available models and algorithms are analyzed in this manuscript, and the main contributions are as follows:

- Two indexes (tracking accuracy and survivability) are introduced to integrally describe system performance in Section 2.
  The current work gives substantial attention to the tracking accuracy. However, the survivability index is seldom discussed. In this work, a definition of survivability is presented and a detailed computation method is also given.
- The optimization models are established for sensor subsets selection for single target and multi-target tracking in Section 3.1. Based on the two performance indexes, two optimization models with multiple constraints are creatively designed. Clearly, the optimization model based on single target tracking is the foundation of multi-target tracking.
- The solutions of the optimization models are also given and the detailed solution steps are clearly given in Section 3.2.

The rest of the paper is organized as follows. The problem formulation—including the introduction of the task network system, system description, and fusion methods—is given in Section 1. Section 2 introduces the two performance indexes: the tracking accuracy and the survivability. In Section 3, the dynamic sensor subsets optimization selection problem—including optimization models and solutions—is studied under the flexible fusion structure. Simulation examples are demonstrated in Section 4. Finally, we conclude the paper in Section 5.

## 2. Problem Formulation

### 2.1. Task Network System

**Figure 1:**
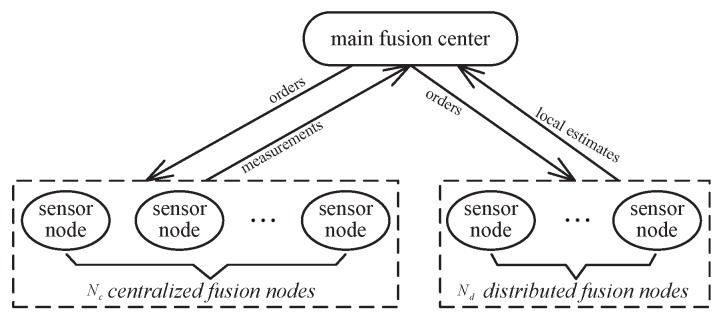
The basic information fusion structure.

A networked information system (NIS) connects all the information units within a given domain in order to construct a real-time and high-speed information system. Information fusion is one of the important techniques used to establish this kind of NIS, and the basic networked information fusion structure is shown in Figure 1. The information fusion center communicates with the local sensor nodes by data link, and then the system fusion center sends commands to each sensor node and receives the measurement data or the local fusion estimation data from sensor nodes. Further global data fusion will be constructed. There are two limitations to this structure:

- Each sensor node can only track a limited number of targets;
- The fusion center can only process a limited amount of sensor measurement data with respect to the limited communication bandwidth and the computing capacity.

The target tracking process is not stable, while the tacking situation is complex, so the system performance indexes are affected by the internal and external unknown factors. In order to achieve an optimal or suboptimal affected system state, we need to regulate the configuration of system surplus resources and change the fusion method of sensor nodes. In the following, we will analyze the target tracking fusion system in detail and design a new system optimization formulation.

### 2.2. System Description and Fusion Methods

The target tracking system includes *M* warplanes (*M* dynamic sensor nodes), and each warplane is equipped with *L* same groups of sensors. B(kbps) denotes the total system communication bandwidth resource. In order to reduce signal caused risk, the system adopts the passive tracking method, with the data receiving period being *T*.

First, we briefly give the nonlinear target tracking system state model as follows:

(1) $$x_{k+1}=f\left(x_{k}\right)+w_{k},$$

(2) $$z_{k,i}=h_{i}\left(x_{k}\right)+v_{k,i},\;\;\;i=1,2,\cdots ,ML,$$

where (1) and (2) are tracking state equation and measurement equation; $x_{k}$ is state vector and $z_{k,i}$ is measurement vector; $f\left(x_{k}\right)$ and $h_{i}\left(x_{k}\right)$ are state transition function matrix and state measurement function matrix, respectively; process noise $w_{k}$ and measurement noise $v_{k,i}$ are zero mean white Gaussian noise with the covariance $Q_{k}$ and $R_{k,i}$, respectively, while the measurement noises between each sensor are uncorrelated.

In this work, the UKF is taken as the basic data filtering method, and the details refer to [13,14,15].

Suppose in time period *t* that there are $m_{c,t}$ and $m_{d,t}$ number of centralized fusion nodes and distributed fusion nodes, respectively, and $m_{c,t}+m_{d,t}\leq M$ (note: subscript notation “*c*” denotes the part of centralized fusion and “*d*” denotes the part of distributed fusion). The active sensor numbers are $n_{i_{c},t},\;\;\;\left(i_{c}=1,2,\cdots ,m_{c,t}\right)$ and $n_{i_{d},t},\;\;\;\left(i_{d}=1,2,\cdots ,m_{d,t}\right)$, and

(3) $$\left\{\begin{array}{c} n_{c,t}=\sum_{i_{c}=1}^{m_{c,t}}n_{i_{c},t}\\ n_{d,t}=\sum_{i_{d}=1}^{m_{d,t}}n_{i_{d},t} \end{array}\right.,\;\mathrm{and}\;n_{c,t}+n_{d,t}\leq M\cdot L.$$

The adopted dimension expansion fusion method and local estimate weighted fusion method for system centralized fusion nodes and distributed fusion nodes are as follows [16]:

- Dimension Expansion Fusion Method
  Integrate $N_{c}$ measurement equations into a large measurement equation
  (4) $$Z_{k}=H\left(x_{k}\right)+V_{k},$$
  and
  $$E\left\{V_{k}\right\}=0,E\left\{V_{k}V_{k}^{T}\right\}=R_{k}^{*},$$
  where
  $$Z_{k}={\left[\begin{array}{ccc} \begin{array}{cc} z_{k,1}^{T} & z_{k,2}^{T} \end{array} & \cdots & z_{k,n_{c,t}}^{T} \end{array}\right]}^{T},$$
  $$H_{k}={\left[\begin{array}{ccc} \begin{array}{cc} h_{1}^{T}\left(x_{k}\right) & h_{2}^{T}\left(x_{k}\right) \end{array} & \cdots & h_{n_{c,t}}^{T}\left(x_{k}\right) \end{array}\right]}^{T},$$
  $$V_{k}={\left[\begin{array}{cc} \begin{array}{ccc} v_{k,1}^{T} & v_{k,2}^{T} & \cdots \end{array} & v_{k,n_{c,t}}^{T} \end{array}\right]}^{T},$$
  $$R_{k}^{*}=\left[\begin{array}{cccc} R_{k,1} & 0 & \cdots & 0\\ 0 & R_{k,2} & \cdots & 0\\ \vdots & \vdots & & \vdots \\ 0 & 0 & \cdots & R_{k,n_{c,t}} \end{array}\right].$$
  Based on the state equation and the measurement equation, applying the basic UKF algorithm, the multi-sensor centralized dimension expansion fusion estimator is
  (5) $$\left\{\begin{array}{c} {\hat{x}}_{k|k}={\hat{x}}_{k|k-1}+K_{k}\left(Z_{k}-{\hat{Z}}_{k|k-1}\right),\\ P_{k|k}=P_{k|k-1}-K_{k}P_{zz,k|k-1}K_{k}^{T} \end{array}\right.$$
  where
  (6) $$\left\{\begin{array}{c} P_{xz,k|k-1}=\sum_{j=0}^{2n}\omega_{cov,j}\left({Ø}_{k|k-1,j}-{\hat{x}}_{k|k-1}\right)\\ \;\;\;\;\;\;\;\;\;\;\;\;\;\;\;\;\;\;\;\;\;\;\;\;\;\;\;\;\;\;\;\;\;\;\;\;\;\;\times {\left(Y_{k|k-1,j}-{\hat{Z}}_{k|k-1}\right)}^{T},\\ P_{zz.k|k-1}=\sum_{j=0}^{2n}\omega_{cov,j}\left(Y_{k|k-1,j}-{\hat{Z}}_{k|k-1}\right)\\ \;\;\;\;\;\;\;\;\;\;\;\;\;\;\;\;\;\;\;\;\;\;\;\;\;\;\;\;\;\;\;\;\;\;\;\;\;\;\;\times {\left(Y_{k|k-1,j}-{\hat{Z}}_{k|k-1}\right)}^{T}+R_{k}^{*},\\ K_{k}=P_{xz,k|k-1}P_{zz,k|k-1}^{-1}. \end{array}\right.$$
- Local Estimate Weighted Fusion Method
  The local estimate weighted fusion estimator is
  (7) $$\left\{\begin{array}{c} {\hat{x}}_{k|k}=\sum_{i_{d}=1}^{m_{d,t}}\frac{P_{k|k,i_{d}}^{-1}}{P_{k|k}^{-1}}{\hat{x}}_{k|k,i_{d}},\\ P_{k|k}^{-1}=\sum_{i_{d}=1}^{m_{d,t}}P_{k|k,i_{d}}^{-1}. \end{array}\right.$$
  From (3) to (6), we can get the state estimate ${\hat{x}}_{k|k,c}$ and the estimate error covariance $P_{k|k,c}$ of the centralized fusion part; from (7), we can get the state estimate ${\hat{x}}_{k|k,d}$ and the estimate error covariance $P_{k|k,d}$ of the distributed fusion part, so the global fusion results of the system are
  (8) $${\hat{x}}_{k|k,sys}=\frac{P_{k|k,c}^{-1}}{P_{k|k,sys}^{-1}}{\hat{x}}_{k|k,c}+\frac{P_{k|k,d}^{-1}}{P_{k|k,sys}^{-1}}{\hat{x}}_{k|k,d},$$
  (9) $$P_{k|k,sys}^{-1}=P_{k|k,c}^{-1}+P_{k|k,d}^{-1}.$$

As we know, there are several ways to exchange data in task network systems. Because this paper considers a kind of special air task network system which is strictly limited to communication among nodes, it only considers a simple approach. Namely, all available sensors send local information to the fusion center; for example, the nodes under the centralized fusion mode send the measurements, and the nodes under the distributed fusion mode send the local estimates. There is no commutation among local available sensors. All fusion operations are done in the fusion center, regardless of the centralized fusion and the distributed fusion. Thereby, the centralized fusion and the distributed fusion are carried out in parallel with the data communication; for example, the group method with multiple CPUs can be used. However, for the fusion process with the centralized and the distributed information in the fusion center, there are several ways to integrate the information. Strictly speaking, the fusion process is not in parallel with the fusion center for the centralized fusion and the distributed fusion. Commonly, the fusion is performed under a given sequential rule, and it is highly effective because the CPU in the fusion center has strong computation ability.

## 3. Analysis of System Performance Indexes

The purpose of this paper is to improve the self-adjustment ability of the target tracking system, and the system optimization goal is to maximize the system performance within certain resources to solve the optimal configuration problem of system resources. Target tracking accuracy and system survivability are the two most important performance judgements of the target tacking fusion system [17]. The system performances have a close relationship with system resources allocation, and the above two indexes will be analyzed qualitatively and quantitatively in detail as follows.

### 3.1. Tracking Accuracy

Accurate target location is the primary task of a target tracking system, which reflects on the tracking accuracy index that to a certain extent determines the system’s overall performance. The tracking accuracy is related to sensor performance, measurement data volume and quality, fusion algorithm and fusion structure, and other external uncontrollable factors. External factors cannot be artificially controlled, so, in order to guarantee the system performance under changing external conditions, we should regulate the deployment and allocation of internal system resources and modify the data fusion method of each sensor node self-adaptively.

Multi-sensor technology improves the system performance to a large degree, but the system becomes more complex. In target tracking, it is necessary to solve the problem of optimal sensor subsets selection. More uncertain sensors lead to better results, and the best way is to choose the optimal combinations of sensors and the optimal combination of fusion methods for each target in order to obtain optimal tracking performance [18,19,20]. Therefore, dynamic sensor management is one of the important links for a sensor network system, while controlling the sensors at optimal working status can greatly improve the system performance.

Compared with distributed fusion, centralized fusion has better fusion accuracy, and in this paper we use the hybrid fusion method to process data based on the UKF. The hybrid fusion method combines the advantages of the centralized and distributed fusion methods, and it is a supplement of those two fusion methods. The system data fusion method is based on the UKF, so the convergence expectation of fusion estimate error covariance can be obtained as the measure standard of target tacking accuracy [21,22,23]. When *M* sensors all adopt the centralized fusion method, the system has the best tracking accuracy; meanwhile, when the system has only one sensor node working, the tacking accuracy is worst.

Suppose the system fusion estimation error covariance is $P_{e,t}$ as calculated by (9). The upper and lower limits of $\mathrm{tr}(P_{e,t})$ are $tr_{a}$ and $tr_{b}$, respectively; i.e.,

$$tr_{a}<\mathrm{tr}\left(P_{e,t}\right)<tr_{b},$$

where

$$\left\{\begin{array}{c} tr_{a}\geq \mathrm{tr}\left(P_{min}\right),\\ tr_{b}\leq \mathrm{tr}\left(P_{max}\right), \end{array}\right.$$

where tr(P) denotes the trace of matrix P; $\mathrm{tr}(P_{min})$ is the convergence expectation of fusion estimation error covariance, while all the sensor nodes adopt the centralized fusion method; $\mathrm{tr}(P_{max})$ is the convergence expectation of fusion estimation error covariance, while the system has only one sensor node working.

### 3.2. System Survivability

The “survivability” index is used to express the possibility that the task system cannot be discovered by the non-cooperative target. The survivability can be used for the nodes and the system. In this paper, it is used for the task network system. This is because the whole network system should be discovered with a large probability once one of the nodes has been found by the non-cooperative target [24]. The factors influencing survivability index include data communication between the network nodes/platforms and the fusion center, radar radiation, etc. For simplicity and considering the passive tracking, we only consider the influence of data communication traffic on the survivability index. For this, the “survivability” is in relation with fusion architecture design. The communication between the fusion center and the nodes with only a passive tracking function is a main event leading to being found for the task network. Thereby, we use the communication time to formulate the survivability of the air task network. Intuitively, longer communication time between the center and the nodes or among nodes means worse survivability for the task network system—namely, they have an inverse relation. In other words, because there is more communication between the fusion center and the local nodes, the fusion center can obtain more available information on the non-cooperative target. Obviously, the fusion accuracy can be improved due to more information. Likewise, more information communication will lead to a greater probability of being detected by non-cooperative targets, namely the risk of being discovered by non-cooperative targets should be increased and the survivability of the tracking system should be reduced. In contrast, less communication means that the information taken by the fusion center is less and the fusion accuracy should be decreased. At the same time, the survivability should be increased because of less communication and the probability of it being detected and discovered should be reduced.

For a given fusion period, the local sensors have several possible samples/measurements. For the centralized fusion nodes, there are many data transmission operations from local sensors to the fusion center in a given fusion period. For the distributed fusion nodes, there is only one transmission operation in a period because many samples can be processed by a local processor to form a unified local estimate, which can be transmitted to the fusion center. If the centralized fusion node is allocated too much in the system structure design, it will bring out too much data transmission traffic, which will have a great influence on system survivability, and our planes will be easily exposed to the nonoperative targets; therefore, we should reduce the number of centralized fusion nodes. In order to guarantee the requirement of tracking accuracy, we have to increase the number of centralized fusion nodes, which leads to a mutual restriction relation between the system survivability index and the tracking accuracy index. Consequently, we should adjust the allocation of the node fusion method under the given conditions in the context of the actual conditions.

In this paper, we consider the survivability index to be mainly determined by the data communication traffic, which can measured by the data communication times $c_{t}$ between local sensor nodes and the fusion center. Here, we do not consider the fully decentralized fusion structure and there is no communication among local sensors. Based on the experience, the survivability index changes little within the limited extent of data transmission times, and with the rapid increase of data transmission times, the survivability index declines quickly. Due to the inverse relation mentioned above, we considered several kinds of decreasing functions. Through graphical simulation analysis, the amplitude–frequency characteristics function of the first order inertia link is comparatively appropriate if it could be properly improved. Accordingly, in terms of background knowledge and experience, two modifications have been done to obtain an available survivability index. The first is to modify the quadratic as a cube, keeping the root sign the same, and the second is to adopt a logarithmic operation to realize dimensionless and standardization effects. The logarithmic form could be considered to be derived from the logarithmic amplitude–frequency characteristics function.

Then, according to the explanation mentioned above, we can get the time computation formula of one communication operation from transmission to reception, which can be expressed by a third-order inertia logarithmic function

(10) $$s_{t}=lg\frac{10}{\sqrt[{3}]{1+\lambda^{3}c_{t}^{3}}},0<s_{t}\leq 1,$$

where 0<λ≤1 is the function attenuation coefficient that determines the function attenuation trend—it is a positive decimal and the λ value is different in different systems. Certainly, other methods for the design of the survivability index may exist, and a comparison study is very important and significant in future work.

In order to realize the sensor management function or flexible fusion structure (namely, to determine the sensor subsets under the centralized and distributed fusion frames), it is important to construct an index to describe the communication time of the whole task network system in a fusion period. This index is taken as the base to optimally solve the sensor subsets. From a normal viewpoint, greater communication time means a greater probability of being discovered by the noncooperative target. Actually, there are many possible ways to construct the system commutation time. Here, we simply take the summation of commination times of all used nodes in a fusion period as the system commutation time. In other words, the system commutation time is composed of two parts, which are: the system centralized fusion part $c_{c,t}=n_{c,t}$ and the system distributed fusion part $c_{d,t}=m_{d,t}$. Then, the system communication time is expressed by

$$c_{sys,t}=c_{c,t}+c_{d,t}=n_{c,t}+m_{d,t}.$$

**Figure 2:**
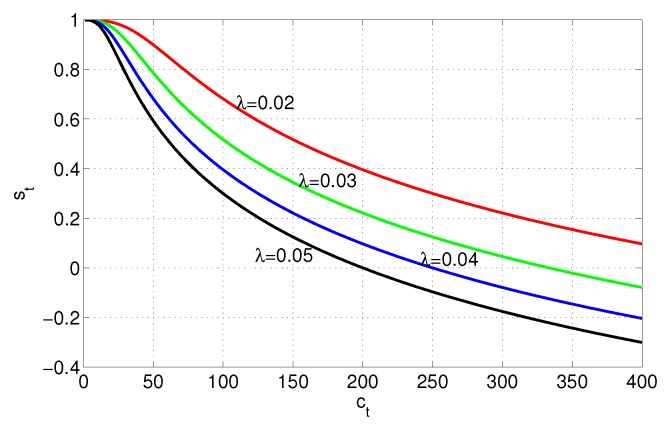
The change curve of *s*.

According to different values of λ, we plot the change curve of $s_{t}$ as Figure 2. As shown in the figure, the change curve of $s_{t}$ basically conforms to the qualitative analysis change requirements of the survivability index, which indicates that the design function of $s_{t}$ is feasible. It should be noted that although there are many ways to design the system communication time index, it is not naturally influenced to establish and solve the optimization model.

It is necessary to consider the security risk factor condition for a single tracking plane, which is an important part of the system survivability index. When the threat of one enemy plane to one of our planes being greater than the safe threshold value, our tracking plane can adopt the action of switching to standby work mode to stop all the sensor activities, and use the avoiding protection method to stay at the tracking formation. Until the enemy threat becomes relatively small, the dormant tracking plane can restart work on the tracking task.

A single-platform security risk coefficient can be treated as the threat value of enemy planes to our planes; a greater threat value means greater mission risk. The method of evaluating threat value was drawn from [25]. Suppose the requirement of system initial risk coefficient safe threshold value is $sr_{0}$, and the real-time evaluation of security risk coefficient is $sr_{i,t},\;\;\;(i=1,2,\cdots ,M$), and

$$sr_{i,t}<sr_{0},\;\;\;i=1,2,\cdots ,M.$$

## 4. Dynamic Sensor Subsets Selection Under Flexible Fusion Structure

### 4.1. Establishment of Optimization Model for Sensor Subsets Selection

Because of the time-varying situation of the task network system induced by moving nodes and non-cooperative target, and limitations such as communication bandwidth and measurement distance, it is necessary to dynamically adjust the system fusion structure, including sensors and fusion methods taking part in the fusion for a given adjustment period. The target tracking accuracy is measured by the estimated error covariance, so the tracking accuracy of this paper is a low-quality index, and it can be standardized by the index quantitative method of cost, defined by the standardized function $P_{p}\left(\mathrm{tr}(P_{e,t})\right)$ as the target tracking accuracy performance value function, and

(11) $$P_{p}\left(\mathrm{tr}(P_{e,t})\right)=\left\{\begin{array}{c} \begin{array}{cc} 1, & \mathrm{tr}\left(P_{e,t}\right)\leq \mathrm{tr}(P_{min}), \end{array}\\ \frac{\mathrm{tr}\left(P_{max}\right)-\mathrm{tr}(P_{e,t})}{\mathrm{tr}\left(P_{max}\right)-\mathrm{tr}\left(P_{min}\right)},\mathrm{tr}\left(P_{min}\right)<\mathrm{tr}\left(P_{e,t}\right)<\mathrm{tr}\left(P_{max}\right),\\ \begin{array}{cc} 0, & \mathrm{tr}\left(P_{e,t}\right)\geq \mathrm{tr}\left(P_{max}\right). \end{array} \end{array}\right.$$

The above formula is a piecewise function which expresses three cases on $P_{e,t}$, $P_{min}$, and $P_{max}$. A larger value of $P_{p}\left(\mathrm{tr}(P_{e,t})\right)$ means the system has better tracking accuracy. The goal of the target tracking system dynamic sensor nodes management is to choose the best combination of the sensor nodes fusion method within the performance index requirement extent, and it has to meet the necessary constraints, which brings the maximum performance into the target tacking system. In summary, we can design a kind of objective optimization model of sensor nodes’ dynamic management:

(12) $$f\left(m_{c,t},m_{d,t}\right)=\arg\;\;\;\max_{f}\;\left(P_{p}\left(\mathrm{tr}P_{e,t}\right)\right),$$

$$s.t.\left\{\begin{array}{c} 0<m_{c,t}+m_{d,t}\leq M,\\ tr_{a}<\mathrm{tr}P_{e,t}<tr_{b},s_{min}<s_{t}\leq 1,\\ sr_{i,t}<sr_{0},\;\;\;r_{i,t}<D_{i},\\ \;\;r_{bw,t}\leq 1, \end{array}\right.$$

where $\;r_{i,t}<D_{i}$ is the constraint of sensor measurement distance, $D_{i}$ is the maximum measurement distance of each sensor node; $r_{bw,t}\leq 1$ is the constraint of data communication bandwidth, $r_{bw,t}$ is the consumption proportion of bandwidth resource,

$$r_{bw,t}=\frac{n_{c,t}\times b+m_{d,t}\times b}{B},$$

where *b* is the occupied communication bandwidth size of one sensor to transmit the measurement data in one time period. The network adjustment principle is shown by optimization formulation Equation (12). The solution of optimization Equation (12) is the numbers and the associated sensors that take part in the centralized and distributed fusion processes.

### 4.2. Multi-Step Solution of Multi-Constraint Optimization Model

The multi-airborne sensor nodes allocation is a problem of multi-target NP combination optimization. For the massive case, with the increase of targets and sensor nodes, it is difficult to solve the model directly, which will cause the problem of “combination explosion”, and it needs a large amount of computation time and storage space, and, given this, it is even possible that the model will remain unsolved. So we adopt the step-by-step solution strategy to gradually reduce the solution space based on the model constraints, and the optimal solution can be obtained.

For the single target system, the solution steps are as follows:

(1) Based on the constraint of sensor node measurement distance, mark off the distant available sensor node subset $S_{1}$;
(2) Check whether the subset $S_{1}$ is consistent with the constraint of single plane security risk to get the security risk available subset $S_{2}$;
(3) Solve all the possible groups of $m_{c,t}$ and $m_{d,t}$ under the constraints of tr(P), *s*, and $r_{bw}$. If $m_{c,t}+m_{d,t}>size\left(S_{2}\right)$, there is no optimization solution, and if the situation is allowable, we can turn back to step (1) or step (2) to widen the constraint extent and proceed to solve the next step; when the $m_{c,t}+m_{d,t}\leq size\left(S_{2}\right)$, the model has solutions, and to get the optimal solution of $m_{c,t}$ and $m_{d,t}$ through the objective function $f\left(m_{c,t},m_{d,t}\right)$;
(4) Allocation of $m_{c,t}$ and $m_{d,t}$ in subset $S_{2}$: firstly to allocate the $m_{c,t}$, the principle of which is to select the sensor nodes that are closer to the fusion center; if there are two sensor nodes whose distances are equidistant, choose the node that has the litter security risk coefficient; then, it is the turn of $m_{d,t}$. Its principle is the same as with $m_{c,t}$, but the allocation range is the remaining sensor nodes of subset $S_{2}$.

For a multi-target system, before the above steps, we need to allocate the optimal sensor node subsets for every target. The allocated sensor nodes of each target should not be more than the average of the total number of nodes for all targets, and every node should be allocated to a target. The allocation principle is to maximize the threat values of airborne planes relative to targets, and the objective optimization model is

(13) $$\delta^{*}\left(t\right)=\arg\;\;\;\min_{\delta }\sum_{j=1}^{N}\sum_{i=1}^{M}\delta_{ij,t}r_{ij,t},$$

$$s.t.\left\{\begin{array}{c} \delta_{ij,t}\in \left\{0,1\right\},\sum_{j=1}^{N}\delta_{ij,t}=1,\\ \left[\frac{M}{N}\right]\leq \sum_{i=1}^{M}\delta_{ij,t}\leq \left[\frac{M}{N}\right]+1, \end{array}\right.$$

where *N* is the number of targets and $\delta_{ij,t}$ is the allocation matrix of sensor node *i* to target *j*. $\delta_{ij,t}=1$ denotes that the sensor node *i* is tracking to the target *j*, otherwise the sensor node *i* is not allocated. $r_{ij,t}$ is the relative distance of the sensor node *i* to the target *j*. So, for the multi-target situation, the objective optimization model of sensor nodes dynamic management is updated as

(14) $$f^{*}\left(m_{c,t}^{j},m_{d,t}^{j}\right)=\arg\;\;\;\max_{f}P_{p}\left(\mathrm{tr}{\left(\sum_{j=1}^{N}{\left(P_{e,t}^{j}\right)}^{-1}\right)}^{-1}\right),$$

$$s.t.\left\{\begin{array}{c} 0<m_{c,t}^{j}+m_{d,t}^{j}\leq \left[\frac{M}{N}\right]+1,\\ tr_{a}^{j}<\mathrm{tr}P_{e,t}^{j}<tr_{b}^{j},s_{min}<s_{t}\leq 1,\\ sr_{ij,t}<sr_{0},\;\;\;r_{ij,t}<D_{i},\\ \;r_{bw,t}\leq 1. \end{array}\right.$$

The $\delta_{ij,t}$ in the multi-target model that can be obtained by using the ant colony optimization algorithm (ACOA) [26,27]. The ACOA is a metaheuristics bionic optimization algorithm that has strong applicability in terms of solving discrete combinatorial problems. In the solution problem of $\delta_{ij,t}$ in this paper, the $\max_{\delta }\sum_{j=1}^{N}\sum_{i=1}^{M}\delta_{ij,t}r_{ij,t}$ can be treated as the elicitation function $\phi_{ij}$, based on the $\sum_{j=1}^{N}\delta_{ij,t}=1$ to setting the tabu table $T_{yes,j}$ of ants’ search targets. The ants firstly randomly generate the target searching sequence, then quickly obtain the sensor node allocation subsets of every target, and finally determine the optimal search path through multi-iteration, and the near-optimal solution of the objective function is obtained.

## 5. Simulation

In order to verify the feasibility of the designed system optimization model, in this section, we demonstrate the simulation in two different situations: one is the single target tracking situation, the other is the multi-target tracking situation. It analyzes the simulation calculation results in these two situations. This paper considers two indexes (tracking accuracy and survivability) to express the system tracking performance. Commonly, this kind of study only uses the tracking accuracy index. Thereby, our scene covers most of the current studies. However, it does not compare the case with only the tracking accuracy index or the case with two indexes in the simulation section.

### 5.1. Single Target Tracking Situation

**Figure 3:**
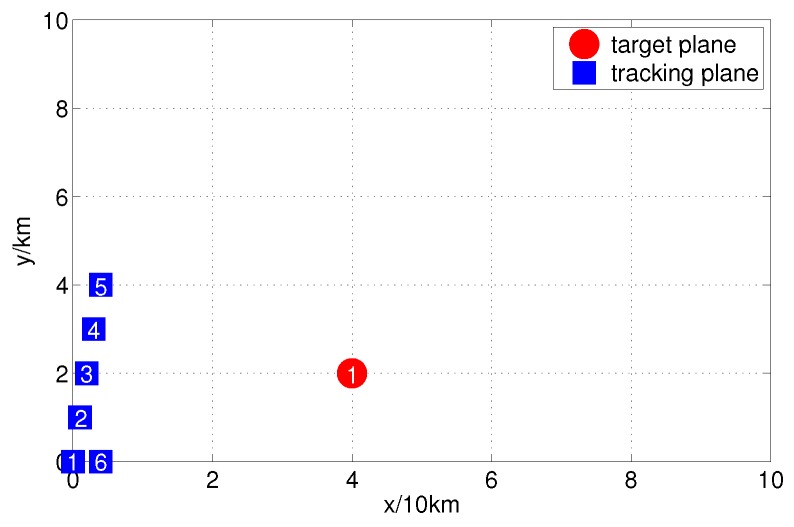
Radar map of enemy and friend initial states.

In time period *t*, our command center sends M=6 reconnaissance planes to track an enemy plane $T_{1}$ that is in our airspace. Assuming the non-cooperative plane has an approximately uniform motion on the X-axis and it has an approximately uniform motion on the Y-axis as well, Figure 3 is the radar map of the enemy’s and our initial states.

The target state is $x={\left[x,y,\dot{x},\dot{y}\right]}^{T}$, its initial state is $x_{0}={\left[50,000,2000,380,120\right]}^{T}$, and $P_{0}=diag\left(64,10,4,4\right)$; the state transfer matrix is

$$F=\left[\begin{array}{cccc} 1 & 0 & T & 0\\ 0 & 1 & 0 & T\\ 0 & 0 & 1 & 0\\ 0 & 0 & 0 & 1 \end{array}\right].$$

The coordinate data of our planes is shown in Figure 3, and the 2th tracking plane is the fusion center. Each plane has been allocated L=3 groups of the same measurement sensors to measure the distance $r_{k}$ and angle $\varphi_{k}$ of the target. In the actual sensor measurement, there will be additive measurement noise $v_{k}$, so in the two-dimensional radar model, the target measurement equation is

$$\begin{array}{c} z_{k,i}=h_{i}\left(x_{k}\right)+v_{k}=\left[\begin{array}{c} r_{k,i}+v_{r,k,i}\\ \varphi_{k,i}+v_{\varphi ,k,i} \end{array}\right]\\ \;=\left[\begin{array}{c} \sqrt{{\left(x_{k}-x_{s,i}\right)}^{2}+{\left(y_{k}-y_{s,i}\right)}^{2}}+v_{r,k,i}\\ tan^{-1}\frac{\left|y_{k}-y_{s,i}\right|}{\left|x_{k}-x_{s,i}\right|}+v_{\varphi ,k,i} \end{array}\right] \end{array},i\in M$$

where $\left(x_{k},y_{k}\right)$ is target coordinate and $\left(x_{s,i},y_{s,i}\right)$ is the *i*-th plane node coordinate.

**Figure 4:**
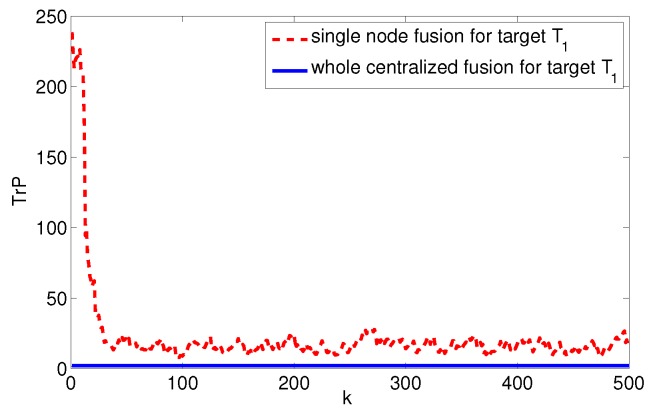
Trace of fusion error covariance of target $T_{1}$.

Assume the system noise $Q_{k}=diag\left(1,1,0.1^{2},0.1^{2}\right)$, the measurement noise $r_{k,i}=diag\left(10^{2},0.1^{2}\right)$, and the measurement period T=0.5. The target data fusion is done with the entirely centralized method and the single node fusion method, respectively, to get the estimated error covariance trace curve of the target state, as shown in Figure 4. So, we can estimate that the upper and lower limits of system fusion tracking accuracy are $E\left[\mathrm{tr}(P_{min})\right]\approx 2.0211$ and $E\left[\mathrm{tr}(P_{max})\right]\approx 16.2603$.

In time period *t*, the performance index requirements and constraint conditions of the system target tracking task are given as follows

$$\left\{\begin{array}{c} 2.5000<\mathrm{tr}P_{e,t}<5.000,\\ 0.95<s_{t}\leq 1,\\ sr_{i,t}<0.50,\;\;\;r_{i,t}<42000, \end{array}\right.$$

By calculating, we can know that all the airborne sensor nodes satisfy the measurement distance constraint and the security risk constraint; however, because of a mechanical failure, plane 3th is out of service. So, $S_{2}=\left\{s_{i}\right\}$ (i≠3) where $s_{i}$ is the *i*-th sensor node.

**Figure 5:**
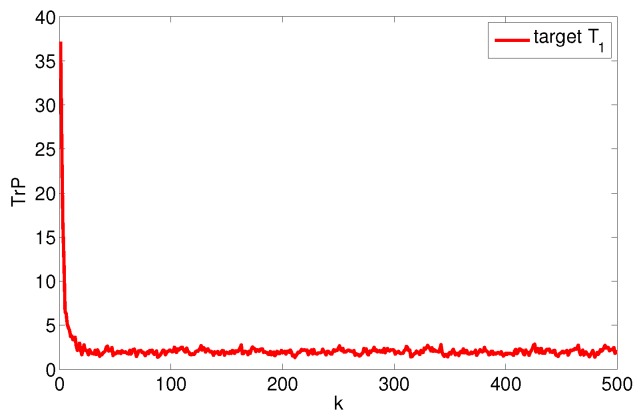
Optimal allocation trace of fusion error covariance of target $T_{1}$.

Based on solution step (3), to circularly verify all the satisfied combinations of $m_{c,t}$ and $m_{d,t}$, and according to the $f\left(m_{c,t},m_{d,t}\right)$, we can select the optimal result $\left(m_{c,t}=2,m_{d,t}=3\right)$. Based on the step (4), we can determine that the optimal allocation options are that the first and second airborne sensor nodes choose the centralized fusion method, and the fourth, fifth, and sixth nodes choose the distributed fusion method. In this allocation option, the trace of the state estimate error covariance of target $T_{1}$ is shown in Figure 5, and we can estimate $E\left[\mathrm{tr}(P_{e,t})\right]\approx 2.6026$, the system survivability index $s_{t}=0.9541$ $\left(\lambda =0.08,c_{t}=9\right)$, and the value of optimization objective function

$$f_{optimal\;\;max}\left(m_{c,t}=2,m_{d,t}=3\right)=0.9592.$$

### 5.2. Multi-Target Tracking Situation

**Figure 6:**
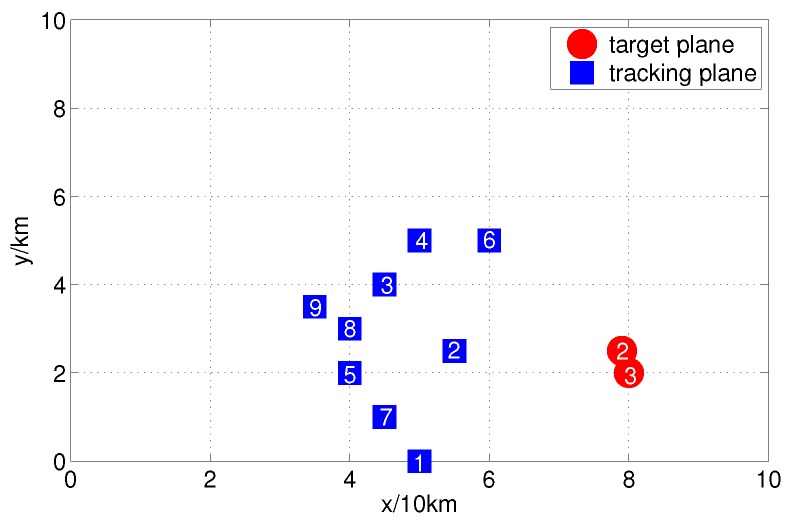
Radar map of enemy and friend instantaneous states.

In time period *t*, our command center sends M=9 reconnaissance planes to track the two enemy planes $T_{2}$ and $T_{3}$ in our airspace. Figure 6 is the radar map of the enemy’s and our instantaneous states. Assume that the non-cooperative planes have approximately uniform accelerated motion on the X-axis and and have approximately uniform accelerated motion on Y-axis as well. The target state vector is $x={\left[x,y,\dot{x},\dot{y},\ddot{x},\ddot{y}\right]}^{T}$, and assuming the target states are $x_{0}^{2}={\left[79000,2500,100,25,2,-2\right]}^{T}$ and $x_{0}^{3}={\left[80000,2000,100,25,2,-2\right]}^{T}$, and $P_{0}^{2,3}=diag\left(100,10,1,1,0.1,0.1\right)$, the state transfer matrix is

$$F=\left[\begin{array}{cccccc} 1 & 0 & T & 0 & \frac{T^{2}}{2} & 0\\ 0 & 1 & 0 & T & 0 & \frac{T^{2}}{2}\\ 0 & 0 & 1 & 0 & T & 0\\ 0 & 0 & 0 & 1 & 0 & T\\ 0 & 0 & 0 & 0 & 1 & 0\\ 0 & 0 & 0 & 0 & 0 & 1 \end{array}\right].$$

**Figure 7:**
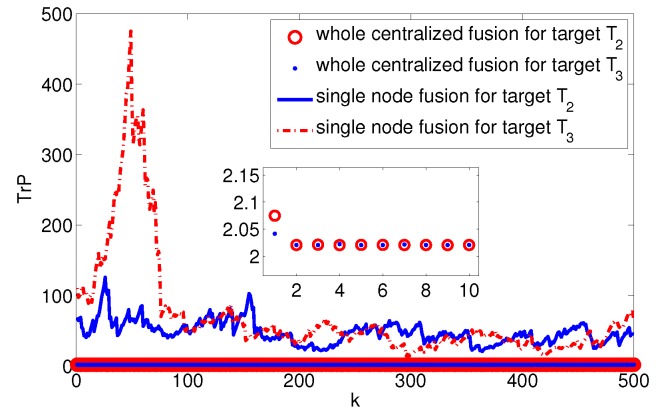
Trace of fusion error covariance of target $T_{2}$ and $T_{3}$.

The coordinate data of our planes is shown in Figure 6, and plane 9 is the fusion center. Each tracking plane has been allocated L=3 groups of the same measurement sensors to measure the distance $r_{k}^{j}$ and angle $\varphi_{k}^{j}$ of targets. In the actual sensor measurement, there will be additive measurement noise $v_{k}^{j}$; so, in the two-dimensional radar model, the target measurement formula is

$$\begin{array}{c} z_{k,i}^{j}=h_{i}\left(x_{k}^{j}\right)+v_{k,i}=\left[\begin{array}{c} r_{k,i}^{j}+v_{r,k,i}^{j}\\ \varphi_{k,i}^{j}+v_{\varphi ,k,i}^{j} \end{array}\right]\\ \;=\left[\begin{array}{c} \sqrt{{\left(x_{k}^{j}-x_{s,i}\right)}^{2}+{\left(y_{k}^{j}-y_{s,i}\right)}^{2}}+v_{r,k,i}^{j}\\ tan^{-1}\frac{\left|y_{k}^{j}-y_{s,i}\right|}{\left|x_{k}^{j}-x_{s,i}\right|}+v_{\varphi ,k,i}^{j} \end{array}\right] \end{array},\begin{array}{c} i\in M,\\ j\in N, \end{array}$$

where $\left(x_{k}^{j},y_{k}^{j}\right)$ is *j*th target coordinate, $\left(x_{s,i},y_{s,i}\right)$ is the *i*th plane node coordinate. Assume the system noise $Q_{k}=diag\left(1,1,0.1^{2},0.1^{2},0.01^{2},0.01^{2}\right)$, the measurement noise $r_{k,i}=diag\left(5^{2},0.1^{2}\right)$, and the measurement period T=0.5. Undertaking the target data fusion with an entirely centralized method and single node fusion method for target $T_{2}$ and $T_{3}$, respectively, we get the estimated error covariance trace curve of the target state as shown in Figure 7. So, we can estimate that the upper and lower limits of system fusion tracking accuracy are $E\left[\mathrm{tr}(P_{min})\right]\approx 2.0207$ and $E\left[\mathrm{tr}(P_{max})\right]\approx 40.0919$.

Based on the above simulation scene, we first allocate $\delta_{ij}$ for $T_{2}$ and $T_{3}$ by using ant colony optimization algorithms, and obtain the optimal security coefficient allocation results $\left(\delta_{32},\delta_{42},\delta_{62},\delta_{82},\delta_{92}\right)$ and $\left(\delta_{13},\delta_{23},\delta_{53},\delta_{73}\right)$. Assume that the performance index requirements and constraint conditions of system target tracking task in time period *t* are

$$\left\{\begin{array}{c} 5.0000<\mathrm{tr}P_{e,t}^{2}<7.0000,6.0000<\mathrm{tr}P_{e,t}^{3}<10.0000,\\ 0.85<s_{t}\leq 1,sr_{ij,t}<0.50,\;\;\;r_{ij,t}<43000. \end{array}\right.$$

Based on solution step (1), we can get

$$\left\{\begin{array}{c} S_{1}^{2}=\left\{\delta_{32},\delta_{42},\delta_{62},\delta_{82}\right\},\\ S_{1}^{3}=\left\{\delta_{13},\delta_{23},\delta_{53},\delta_{73}\right\}. \end{array}\right.$$

Because $sr_{16,t}=0.546>0.50$, the sixth node does not satisfy the security risk constraint, so the available security risk subset is

$$\left\{\begin{array}{c} S_{2}^{2}=\left\{\delta_{32},\delta_{42},\delta_{82}\right\},\\ S_{2}^{3}=\left\{\delta_{13},\delta_{23},\delta_{53},\delta_{73}\right\}. \end{array}\right.$$

Based on solution step (3), to circularly verify all the satisfied combinations of $m_{c,t}^{j}$ and $m_{d,t}^{j}$, and according to the four designed objective optimization function $f^{*}\left(m_{c,t}^{j},m_{d,t}^{j}\right)$, we can determine that the optimal combinations are $\left(m_{c,t}^{2}=2,m_{d,t}^{2}=1\right)$ and $\left(m_{c,t}^{3}=1,m_{d,t}^{3}=3\right)$.

Lastly, according to the above results and solution step (4), we can obtain the optimal airborne sensor nodes allocation results

$$\left\{\delta_{32,c},\delta_{42,d},\delta_{82,c}\right\},\left\{\delta_{13,d},\delta_{23,d},\delta_{53,c},\delta_{73,d}\right\}.$$

**Figure 8:**
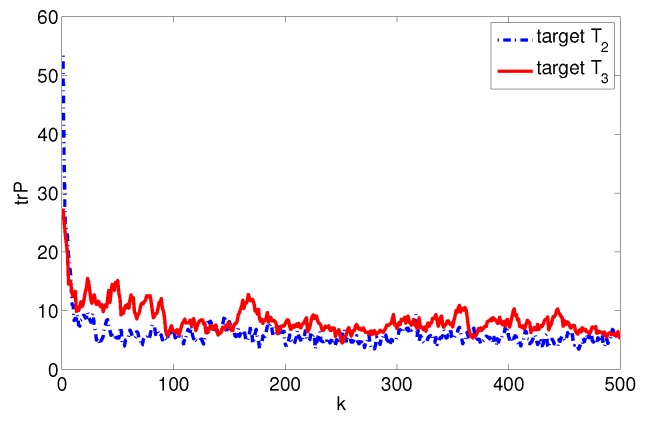
Optimal allocation trace of fusion error covariance of target $T_{2}$ and $T_{3}$.

The estimated error covariance traces of target $T_{2}$ and $T_{3}$ are shown in Figure 8 in the condition of the above allocation options, the estimated values $E\left[\mathrm{tr}P_{e,t}^{2}\right]\approx 5.6625$ and $E\left[\mathrm{tr}P_{e,t}^{3}\right]\approx 7.1698$, the system survivability index $s_{t}=0.8909$ $\left(\lambda =0.08,c_{t}=13\right)$, and the value of optimization objective function $f_{optimal\;\;max}^{*}\left(m_{c,t}^{2,3},m_{d,t}^{2,3}\right)=0.9769$.

### 5.3. Analysis of Simulation Results

For the above two different simulation situations, we achieved the optimal allocation of the multi-sensor target tracking fusion system with the optimization model designed in this paper. In time period *t*, based on the real-time system performance requirements, the optimal number of sensor and optimal fusion method combinations are determined to maximize the system tracking accuracy, and it satisfies the system survivability constraint and other necessary constraints, which can achieve the system self-adaptive performance optimization adjustment function and improve the self-adaptive adjustment ability of the distributed tracking fusion system.

The optimization model in this paper is designed for the problem of system performance instability caused by the changing situations and emergencies in the tracking process. In simulation 1, the system encountered the node failure problem, and in simulation 2, the system encountered the problem of node measurement distance and the security risk of a single airborne plane. From the results of simulation 1 and simulation 2, we can see that the systems have self-adjusting abilities; when the system encounters the above problems and other issues, the system can reject the troublesome nodes and recombine the remaining system resources to optimally allocate, which leads to the stable performance and great anti-interference ability of the target tracking fusion system.

## 6. Conclusions

For the problems of a complex motion model in target tracking process and the changeable motion situations which lead to the instability of tracking systems, this paper studies the flexible fusion structure algorithms and designs a kind of flexible fusion optimization model. The multi-step solution strategy and ant colony optimization algorithm are used to solve the designed model, which can obtain the optimal sensor subsets selection dynamically. By the simulation verification, the designed system optimization model was proven to be feasible and effective, and could improve the self-adjustment ability of a target tracking fusion system and guarantee that the multi-plane cooperative tracking task is accomplished successfully.
